# A porcine reproductive and respiratory syndrome virus (PRRSV)-specific IgM as a novel adjuvant for an inactivated PRRSV vaccine improves protection efficiency and enhances cell-mediated immunity against heterologous PRRSV challenge

**DOI:** 10.1186/s13567-022-01082-5

**Published:** 2022-08-19

**Authors:** Rui Chen, Bing Liu, Xiangmei Zhang, Mengmeng Qin, Jianhui Dong, Guoqian Gu, Chunyan Wu, Jingyu Wang, Yuchen Nan

**Affiliations:** 1 Department of Preventive Veterinary Medicine, College of Veterinary Medicine, Northwest A&F University, Yangling, Shaanxi China; 2 Department of Infectious Diseases and Public Health, Jockey Club College of Veterinary Medicine, City University of Hong Kong, Hong Kong SAR, China

**Keywords:** PRRSV, inactivated PRRSV vaccine, broadly neutralizing antibody, monoclonal antibody, IgM, vaccine adjuvant

## Abstract

Current strategies for porcine reproductive and respiratory syndrome (PRRS) control are inadequate and mainly restricted to immunization using different PRRS virus (PPRSV) vaccines. Although there are no safety concerns, the poor performance of inactivated PRRSV vaccines has restricted their practical application. In this research, we employed the novel PRRSV-specific IgM monoclonal antibody (Mab)-PR5nf1 as a vaccine adjuvant for the formulation of a cocktail composed of inactivated PRRSV (KIV) and Mab-PR5nf1 along with a normal adjuvant to enhance PRRSV-KIV vaccine-mediated protection and further compared it with a normal KIV vaccine and modified live virus vaccine (MLV). After challenge with highly pathogenic (HP)-PRRSV, our results suggested that the overall survival rate (OSR) and cell-mediated immunity (CMI), as determined by serum IFN-γ quantification and IFN-γ ELISpot assay, were significantly improved by adding PRRSV-specific IgM to the PRRSV-KIV vaccine. It was also notable that both the OSR and CMI in the Mab-PR5nf1-adjuvanted KIV group were even higher than those in the MLV group, whereas the CMI response is normally poorly evoked by KIV vaccines or subunit vaccines. Compared with those in piglets immunized with the normal KIV vaccine, viral shedding and serum neutralizing antibody levels were also improved, and reduced viral shedding appeared to be a result of enhanced CMI caused by the inclusion of IgM as an adjuvant. In conclusion, our data provide not only a new formula for the development of an effective PRRSV-KIV vaccine for practical use but also a novel method for improving antigen-specific CMI induction by inactivated vaccines and subunit vaccines.

**Supplementary Information:**

The online version contains supplementary material available at 10.1186/s13567-022-01082-5.

## Introduction

Porcine reproductive and respiratory syndrome virus (PRRSV) is an enveloped positive-strand RNA virus that is classified into the genus *Porartevirus* [1, 2], family *Arteriviridae*, and order *Nidovirales* [3]. Since its discovery in the late 1980s, PRRSV has been recognized as one of the most notorious viruses for the pork industry worldwide and the cause of tremendous economic losses each year [4, 5]. The genome of PRRSV is approximately 15k nucleotides in size and contains at least 10 open reading frames (ORFs) [3]. ORF1a and ORF1b of PRRSV account for three-fourths of the entire PRRSV genome and encode nonstructural proteins (nsps) essential for PRRSV RNA replication, whereas ORFs 2–7 encode PRRSV structural proteins (SPs) required for viral particle assembly [3]. Currently, there are two known species of PRRSV, *Betaarterivirus suid* 1 (formally designated *PRRSV-1*) and *Betaarterivirus suid* 2 (formally designated *PRRSV-2*) [6–8]. These two PRRSV species are serotypically distinct and share only approximately 60% nucleotide sequence similarity [6–8], whereas the overall disease phenotype, gross clinical signs, and genomic organization are similar between them [9].

Current strategies for PRRS control are inadequate despite substantial efforts dedicated to developing an effective PRRSV vaccine [10]. Although the first modified live virus (MLV) vaccine has been commercially available on the market for over two decades, the prevalence of PRRSV infection in swine herds remains high [11]. It is generally considered that PRRSV-MLVs confer late but effective protection against challenge by genetically homologous PRRSV strains but only partial or no protection against heterologous strains [12, 13], which is consistent with observations regarding atypical PRRS outbreaks in MLV-vaccinated herds [14, 15]. Moreover, it is notable that week-long viremia with the vaccine virus persisted in MLV-immunized piglets, which could lead to transmission of the vaccine virus to naive animals [12, 16], thereby raising concerns that PRRSV-MLVs might revert to virulence or recombine with wild-type field strains, with frequent occurrences of both types of events previously reported [17–20].

Due to these safety concerns for PRRSV-MLVs, inactivated PRRSV vaccines (KIVs) have been licenced only in China and were previously licenced in the United States. However, since 2005, KIVs have no longer been marketed in the US due to their poor performance [12]. Several reports have explained that the poor protection afforded by PRRSV-KIVs is caused by failure to elicit the production of detectable PRRSV-specific neutralizing antibodies (NA) and cell-mediated immunity (CMI) after KIV immunization [21–23]. Nevertheless, clinical practice has also suggested that long-term application of PRRSV-KIVs conferred benefits in PRRSV-circulating swine herds, such as promoting the production of PRRSV-specific antibodies and CMI responses against circulating viruses [22, 23]. This is consistent with a report that repeated exposure or long-term administration of PRRSV-KIV in seropositive swineherds boosts anti-PRRSV immunity and yields a significant improvement in reproductive performance [24]. Therefore, several attempts have been made to improve the immunity induced by KIVs, such as intranasal delivery of a nanoparticle-entrapped KIV along with poly(lactic-co-glycolic) acid or whole-cell lysate of *Mycobacterium tuberculosis* as an adjuvant that elicits broadly cross-protective anti-PRRSV immunity against heterologous PRRSV strains [25, 26]. These reports suggested that a special formulation (nanoparticles) combined with the novel adjuvant poly(lactic-co-glycolic) acid or *M. tuberculosis* lysate may enhance the immune response evoked by PRRSV-KIVs during immunization.

In our previous report, we identified the novel IgM-type monoclonal antibody (Mab)-PR5nf1 that is capable of neutralizing all tested PRRSV isolates of both *PRRSV-1* and *PRRSV-2* in vitro [27]. Although compared with IgG, IgM is not suitable for application as a treatment in vivo, immunization of mice with inactivated PRRSV and Mab-PR5nf1 with a normal vaccine adjuvant enhanced CMI. In this study, we immunized piglets with Mab-PR5nf1-adjuvanted inactivated PRRSV to investigate whether IgM complexed with PRRSV-KIV could improve the protection efficiency of PRRSV-KIV against heterologous highly pathogenic (HP)-PRRSV. Our data suggested that immunization with IgM-adjuvanted PRRSV-KIV twice improved protection efficiency against heterologous HP-PRRSV challenge in piglets compared with that in piglets immunized with KIV alone or MLV.

## Materials and methods

### Cell and viruses

PRRSV-permissive MARC-145 cells were maintained in Dulbecco’s modified Eagle medium (DMEM) (Thermo Fisher Scientific, Waltham, MA, USA) supplemented with 10% FBS (Thermo Fisher Scientific). The development of the immortalized porcine alveolar macrophage (PAM) cell line 3D4/21 (ATCC^®^CRL-2843™) with stable expression of porcine CD163 (CRL-2843^CD163^) permissive for PRRSV was previously described [28]. To further increase the susceptibility of CRL-2843^CD163^ cells to PRRSV to mimic natural PAM susceptibility, the full ORF of porcine CD169 was artificially synthesized and ligated to the pLVX lentiviral vector to introduce porcine CD169 into CRL-2843^CD163^ cells to generate CRL-2843^CD163/CD169^ cells. CRL-2843^CD163/CD169^ cells were maintained in DMEM (Thermo Fisher Scientific) supplemented with 10% FBS (Thermo Fisher Scientific) as described for MARC-145 cells.

The PRRSV strains used in this study were VR2332 (GenBank: EF536003.1) and HP-PRRSV isolate XJA1 [shares the highest homology with HP-PRRSV-JXA1 (GenBank: EF112445.1)]. Based on sequence analysis, HP-PRRSV-JXA1 shared approximately 87% nucleotide similarity with VR2332. A licenced MLV, Ingelvac PRRS MLV (herein named MLV), was kindly provided by the local distributor of Boehringer-Ingelheim in China. All PRRSV isolates were used to inoculate MARC-145 cells. The median tissue culture infectious dose (TCID_50_) of all PRRSV isolates was titrated in MARC-145 cells as previously described [29].

### Ethics statement and animal studies

The animal protocol was reviewed and approved by the Animal Welfare Committee of Northwest A&F University. All animals were monitored daily for any clinical signs during the whole experiment. Briefly, four-week-old piglets were obtained from a PRRSV-free pig farm near Yangling, Shannxi, and further screened for CSFV, PRRSV, PCV2 and ASFV and for corresponding antibodies. Only piglets (*n* = 25) negative for all examined pathogens and antibodies against PRRSV and ASFV were selected for this study. The piglets were randomly divided into five groups (*n* = 5) and housed in separate rooms. Details about the piglet groupings are provided in Table 1.

**Table 1:** **Animal groups**

Group Name	Vaccine used for immunization	Virus used for Challenge
MOCK	PBS	PBS
HP-PRRSV	PBS	HP-PRRSV-XJA1
MLV/HP-PRRSV	MLV	HP-PRRSV-XJA1
KIV/HP-PRRSV	100 μg of Inactivated PRRSV-VR2332*	HP-PRRSV-XJA1
IgM + KIV/HP-PRRSV	IgM Mab-PR5nf1(1 mg) + 100 μg of inactivated PRRSV-VR2332*	HP-PRRSV-XJA1

^*^Immunization twice at an interval of 2 weeks

### Antibody production and purification

Hybridomas secreting the PRRSV-specific broadly neutralizing IgM Mab-PR5nf1 were maintained in RPMI 1640 medium (Biological Industries) supplemented with 10% FBS, and IgM was purified from cell culture supernatant as previously described [27]. Briefly, cell culture supernatants were harvested by centrifugation at 7000 ×*g* for 15 min to remove hybridomas. Next, the supernatant was concentrated using a Labscale TFF System (EMD Millipore, Boston, MA, USA) using a filter with a 100-kDa molecular mass cut-off. Mab from concentrated supernatant was precipitated using saturated ammonium sulfate followed by centrifugation at 12 000 × *g* for 30 min to collect the protein pellet. After the pellet was resuspended in 200 mM Tris–HCl (pH 8.0), followed by dialysis in 20 mM Tris–HCl (pH 8.0) to remove serum cell culture medium proteins, IgM was collected by centrifugation at 12 000 × *g* for 10 min at 4 °C and further purified using protein L (Genscript, Nanjing, China)-based affinity purification. Purified IgM was concentrated to the indicated concentrations using 100-kDa cut-off ultrafiltration centrifugal tubes (EMD Millipore) and quantified using a BCA protein quantification kit (Thermo Fisher Scientific).

For the generation of rabbit polyclonal antibodies against truncated porcine CD163 and CD169, the cDNA sequences of truncated CD163 (scavenger receptor cysteine-rich domains 1–5) and CD169 (aa 1–540) were artificially synthesized and ligated into pET-28a vectors infused with a C-terminal 8X His tag, followed by transformation into *E. coli* strain BL21 (DE3), and cultured in LB medium at 37 °C until the cells were induced with 0.5 mM isopropyl β-D-thiogalactoside (IPTG) at 25 °C. After IPTG induction, bacterial cells were collected and resuspended in cell lysis buffer (50 mM Tris–HCl [pH 7.5], 150 mM NaCl, 1 mM EDTA, 1 mM AEBSF [4-benzenesulfonyl fluoride hydrochloride], and 5% glycerol) for sonication. Next, inclusion bodies containing recombinant proteins were washed with phosphate-buffered saline (PBS) and reconstituted with 8 M urea (Sigma‒Aldrich, St. Louis, MO, USA) for Ni + affinity chromatography purification (Transgene, Beijing, China). Recombinant protein was eluted from Ni + agarose using PBS buffered 8 M urea solution containing 150 mM imidazole. Dialysis of recombinant proteins was conducted by a gradient decrease in urea concentration until the buffer was completely replaced by PBS or until the minimum urea concentration without visible protein precipitations during dialysis was reached, and the resultant protein was quantified by a BCA protein assay kit (Thermo Fisher Scientific). Immunization of rabbits and purification of antibodies against CD163 and CD169 were conducted by Genscript Co., Ltd.

### Immunization and challenge of piglets

Purification of PRRSV-VR2332 virions was performed as previously described [28]. Purified PRRSV virions were inactivated using 0.1% β-propiolactone and incubated at 4 °C overnight, followed by a 2-h incubation at 37 °C to allow degradation of β-propiolactone. For each piglet, 100 μg of purified virus was used for immunization. Briefly, 100 μg of inactivated virus was mixed with 1 mg of Mab-PR5nf1 in a 1 mL volume of PBS and further incubated for another 2 h to form the IgM immune complex (IgM-IC). Next, IgM-IC (KIV + IgM) or 100 μg of inactivated virus (KIV) was emulsified in Montanide™ ISA 206 water-in-oil adjuvant (Seppic, Paris, France) at a ratio of 46:54 for immunization. Piglets were immunized with KIV or KIV + IgM twice at a two-week interval. Piglets immunized with MLV (single-dose immunization, 1 × 10^6^ TCID_50_ in 1 mL per piglet) at the same time as the first KIV immunization were included as positive controls. Three weeks after the second immunization, all piglets except those in the negative control group were challenged with HP-PRRSV-XJA1.

### Pathological examination

To evaluate the protective efficacy of different vaccine candidates against HP-PRRSV challenge, the lungs of all piglets were examined for gross pathological changes immediately after death or after euthanasia of the remaining survivors at 21 days post-infection (dpi). Samples of lung tissue were collected and fixed by immersion in 10% neutral buffered formalin. Next, fixed samples were embedded in paraffin followed by sectioning for use in histological examination. Sections were stained with haematoxylin and eosin (H&E) to improve the detection of micropathological changes.

### RNA isolation and quantitative real-time PCR (qPCR)

Total RNA was extracted from serum samples, nasal swabs and PAMs using TRIzol reagent (Thermo Fisher Scientific) in accordance with the manufacturer’s instructions. PRRSV RNA detection via qPCR was conducted using RealPCR PRRSV-2 RNA Mix (IDEXX, Westbrook, Maine, USA) according to the manufacturer’s instructions. The manufacturer’s cut-off Ct value of 38 was set for analysis of qPCR data that reflected PRRSV RNA levels. For qPCR-based evaluation of the relative expression of target genes, total RNA was extracted from PAMs using TRIzol Reagent (Thermo Fisher Scientific) in accordance with the manufacturer’s instructions. To evaluate the relative gene expression of PAMs, reverse transcription and qPCR were conducted using a PrimeScript RT Reagent Kit (TaKaRa, Dalian, China) and 2 × RealStar Power SYBR Mixture (Genstar, Beijing, China) according to the manufacturer’s instructions. Transcripts of GAPDH were also amplified in parallel for use in normalizing total RNA input. Relative quantification of target genes was calculated using the 2^−ΔΔCt^ method. The sequences of the primers used in this study are listed in Table 2.

**Table 2:** **List of primers and corresponding sequences used in this study**

Primer	Sequence (5'-3')	Description
ISG15-F	GCAAAGCTTCAGAGACCCAC	qPCR for ISG15 mRNA
ISG15-R	GCCAGACCTCATAGGCGTTG	
pGM-CSF-F2	ATGCCATCAAAGAAGCCCTGA	qPCR for pGM-CSF mRNA
pGM-CSF-R2	GCTTGTACAGGTTCAGGCGA	
CCL2-F	GCAAGTGTCCTAAAGAAGCAGTG	qPCR for CCL2 mRNA
CCL2-R	TCCAGGTGGCTTATGGAGTC	
CXCL10-F	TGCCCACATGTTGAGATCAT	qPCR for CXCL10 mRNA
CXCL10-R	CGGCCCATCCTTATCAGTAG	
GAPDH-F	CCTTCCGTGTCCCTACTGCCAAC	qPCR for GAPDH mRNA
GAPDH-R	GACGCCTGCTTCACCACCTTCT	
iNOS-F	GCACCTGCGTTATGCCACCAAC	qPCR for iNOS mRNA
iNOS-R	TGAGCTGAGCGTTCCAGACCC	
IFN-γ-F	TCACTGATGGCTTTGCGCTG	qPCR for IFN-γ mRNA
IFN-γ-R	AGAGCATGATCCGAGACGTG	
TNF-α-F	AGAGCATGATCCGAGACGTG	qPCR for TNF-α mRNA
TNF-α-F	CAGTAGGCAGAAGAGCGTGG	
IL-6-F	ACAAAGCCACCACCCCTAAC	qPCR for IL-6 mRNA
IL-6-R	CGTGGACGGCATCAATCTCA	
CD163-F	TCCTTGTGGGATTGTCCTGC	qPCR for CD163 mRNA
CD163-F	AGGGATTCTCGGCTCTTTGC	
IL-4-F	CTTCGGCACATCTACAGACACC	qPCR for IL-4 mRNA
IL-4-R	CTTCATAATCGTCTTTAGCCTTTCC	
IL-8F	TCCTGCTTTCTGCAGCTCTC	qPCR for IL-8 mRNA
IL-8R	GGGTGGAAAGGTGTGGAATG	
IL-13-F	GGTCAATATCACCCAGAACCAGAAG	qPCR for IL-13 mRNA
IL-13-R	TGCAGTCGGAGATGTTGATGAGG	
IL-12-F	TACCACTTGAACTAGCCACGAAT	qPCR for IL-12 mRNA
IL-12-R	CTAAGGCACAGGGTTGTCATAAA	
CCL17-F	ATGCAGCTCGAGGAACCAAC	qPCR for CCL17 mRNA
CCL17-R	GTCACAAGCACAATGGCGTC	
IL-1β-F	GACCCCAAAAGATACCCAAA	qPCR for IL-1β mRNA
IL-1β-R	TCTGCTTGAGAGGTGCTGATG	
TGF-β-F	TCCAAGGACCCTTCTCGGAT	qPCR for TGF-β mRNA
TGF-β-R	AAAAACCGAGATGGGCGAGA	
MGL-1-F	ACTTCTCCGGCATGGTTCTG	qPCR for MGL-1 mRNA
MGL-1-R	GTTGAGCACTTTCGCAGCAA	
IRF-4-F	CCGTCATTAGTGCGTCAGTTCT	qPCR for IRF-4 mRNA
IRF-4-R	TTGCAGCCCACAAAAAGCA	
IL-10-F	CGGCGCTGTCATCAATTTCTG	qPCR for IL-10 mRNA
IL-10-R	CCCCTCTCTTGGAGCTTGCTA	

### Enzyme-linked immunosorbent assay (ELISA)

Sequential serum samples harvested at the indicated time points from all experimental animals before HP-PRRSV challenge were screened for anti-PRRSV antibodies using an IDEXX HerdChek PRRS X3 ELISA Kit (IDEXX) according to the manufacturer’s instructions. The IFN-γ level of sequential serum samples was determined using an IFN-γ ELISA Kit (Thermo Fisher Scientific) according to the manufacturer’s instructions.

For the evaluation of serum antibodies against different PRRSV structural proteins, recombinant expression of HP-PRRSV-GP5 ectodomain and N protein were conducted as previously described [30], whereas all other PRRSV structural proteins, including GP2a, GP3, GP4 and M, were cloned from the cDNA of the PRRSV-JXA1 strain and then ligated to the pET-28a vector infused with a C-terminal 8XHis tag, followed by transformation into *E. coli* strain BL21 (DE3) and culturing in LB medium at 37 °C until cells were induced with 0.5 mM isopropyl β-D-thiogalactoside (IPTG) at 25 °C. After IPTG induction, bacterial cells were collected and resuspended in cell lysis buffer (50 mM Tris–HCl [pH 7.5], 150 mM NaCl, 1 mM EDTA, 1 mM AEBSF [4-benzenesulfonyl fluoride hydrochloride], and 5% glycerol) for sonication, after which inclusion bodies containing recombinant proteins were washed with PBS and reconstituted with 8 M urea (Sigma‒Aldrich) for Ni + affinity chromatography purification (Transgene). Recombinant proteins were eluted from Ni + agarose using PBS buffered 8 M urea solution containing 50 to 200 mM imidazole. Dialysis of recombinant 6 × His-PRRSV-recombinant proteins was conducted by a gradient decrease in urea concentration until the buffer was completely replaced by PBS or the minimum urea concentration without visible protein precipitations during dialysis was reached. All recombinant proteins were quantified by a BCA protein assay kit (Thermo Fisher Scientific). Next, recombinant proteins (400 ng) were used to coat 96-well polystyrene microplates (Corning Inc. Corning, NY, USA) in a 100 μL volume of PBS (pH 8.0) overnight at 4 °C. Plates were further blocked with 5% skim milk in PBS containing 0.5% Tween 20 (Sigma‒Aldrich). Diluted serum was added to the wells and incubated for one hour at 37 °C, followed by washing with PBS containing 0.5% Triton X-100 (Sigma‒Aldrich). The binding of antibodies to the corresponding antigen was detected using HRP-conjugated goat anti-swine IgG antibodies (Jackson ImmunoResearch, West Grove, PA, USA) and visualized by a TMB substrate kit (Tiangen Biotech, Beijing, China). The absorbance of each well was measured using a Victor ™ X5 Multilabel Plate Reader (Perkin Elmer, Waltham, MA, USA) at 450 nm.

### Virus neutralization assay

Virus neutralization assays were carried out using MARC-145 cells and CRL-2843^CD163/CD169^ cells based on the ability of PRRSV-neutralizing antibodies in serum samples to block infection as previously described, with modification [31, 32]. Briefly, serum was heat-inactivated at 56 °C for 30 min. Next, serum samples were diluted to create twofold serial dilutions. To each dilution, an equal volume of HP-PRRSV at 0.2 MOI was added, followed by incubation of the mixtures at 37 °C for 1 h to allow antibodies to bind with PRRSV virions. After incubation, the mixtures were transferred to MARC-145 or CRL-2843^CD163/CD169^ monolayers in 24-well plates and incubated for an additional 17 h at 37 ℃ before the cells were fixed for immunofluorescence assays. Fluorescent foci were counted to quantify the infection of cells by PRRSV. Compared to that of the antibody samples from mock-infected pigs, the maximum dilution titre of the serum sample that reduced PRRSV replication by 50% or more (as counted by fluorescent foci) was counted as the virus neutralization titre.

### Immunofluorescence assay (IFA)

Cells with the indicated treatment were fixed in 4% paraformaldehyde (Sigma‒Aldrich) and permeabilized with PBS containing 0.5% Triton X-100 (Sigma‒Aldrich). IFAs were carried out using the PRRSV N-specific Mab PP7EF11. Specific interactions between Mab-PP7EF11 and the target were detected using an Alexa Fluor^®^555-labelled goat anti-mouse secondary antibody (Thermo Fisher Scientific). Cell nuclei were visualized using DAPI (Thermo Fisher Scientific), and IFA samples were observed under a Leica DM1000 fluorescence microscope (Leica, Germany).

### Porcine IFN-γ enzyme-linked immunospot assay (ELIspot)

Peripheral blood mononuclear cells (PBMCs) were isolated from piglets using Ficoll-Paque PLUS (GE Healthcare, Boston, MA, USA) according to the manufacturer’s instructions. Next, 1 × 10^4^ PBMCs were added to 96-well plates before adding 10 μg of recombinant PRRSV N proteins, and then the plates were incubated for cell stimulation and proliferation. The cells were analysed for IFN-γ production using a Porcine IFN-γ ELISpot PLUS Kit (Mabtech, Cincinnati, OH, USA) according to the manufacturer’s instructions. The image and number of dots in each well were recorded using an Autoimmun Diagnostika GmbH EliSPot Reader System (Advanced Imaging Devices GmbH, Strassberg, Germany).

### Statistical analysis

A post hoc statistical analysis for the pairwise comparisons of serum IFN-γ levels and number of IFN-γ-producing cells among PBMCs was performed using GraphPad Prism version 5.0 (GraphPad Software, San Diego, CA, USA). Differences in indicators between experimental groups and controls were assessed using Student’s *t* test or one-way analysis of variance (ANOVA) for comparisons among more than two groups. A two-tailed *P* value < 0.05 was considered statistically significant.

## Results

### Experimental design and immunization schedules

**Figure 1:**
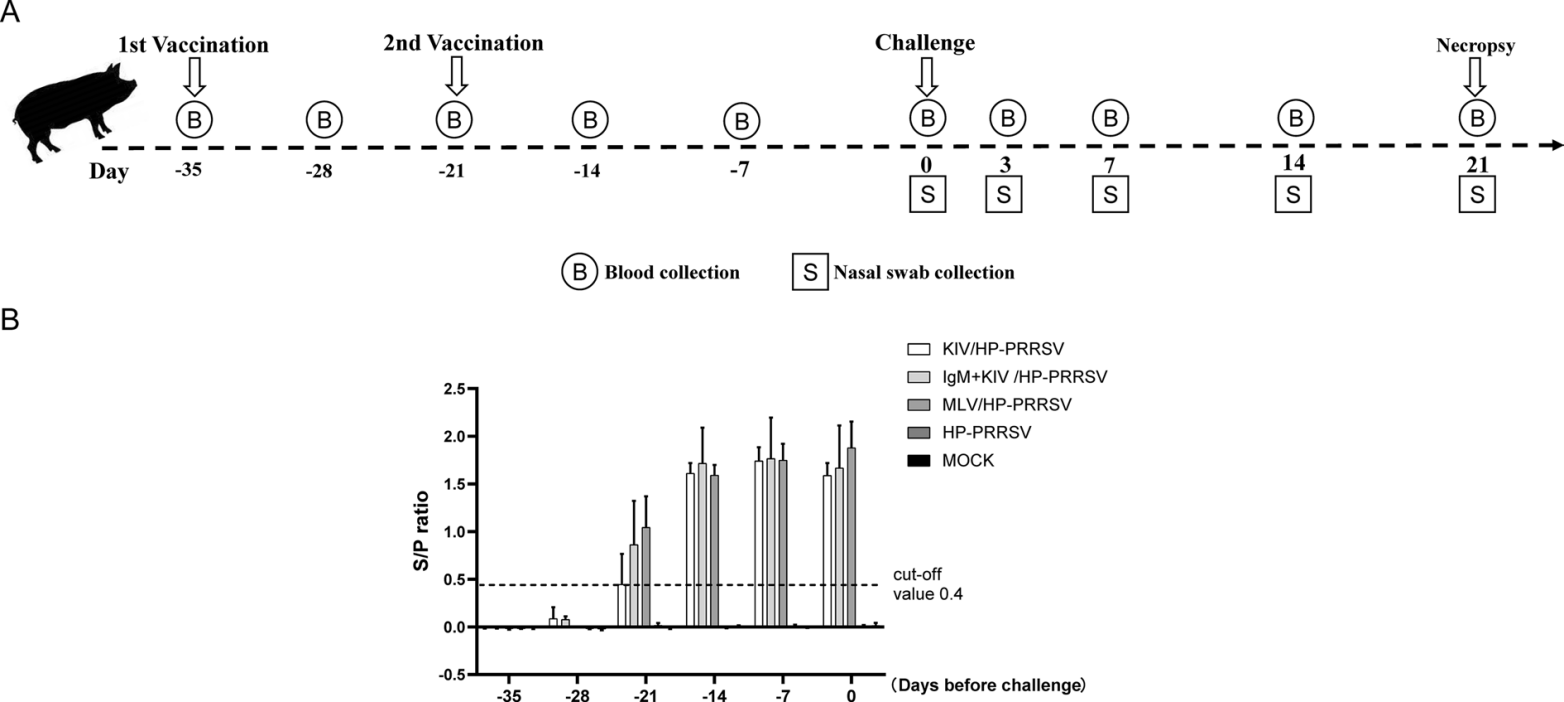
**Schematic illustration of the experimental protocol and ELISA of seroconversion prior to HP-PRRSV challenge. A.** After piglets were housed, different vaccines (MLV, KIV, and IgM + KIV) were used to vaccinate animals via the intramuscular route using the indicated doses described in the Methods section. Blood and nasal swab samples were collected at the indicated times, and all surviving animals were necropsied at 21 dpi. **B.** After immunization of piglets with different vaccines, serum samples were collected weekly and examined to detect seroconversion using an IDEXX HerdChek PRRS X3 ELISA kit. All experiments were repeated at least three times for each serum sample. Data are expressed as the mean ± SD.

In our previous research investigating the broad neutralization activity of Mab-PR5nf1 against heterologous PRRSV isolates, a preliminary experiment using 100 μg of Mab-PR5nf1 as a vaccine adjuvant for PRRSV-KIV (SD16 strain, 10 μg of purified virion) immunization of mice was conducted [27]. PRRSV-specific IFN-γ ELISpot assay for CD8^+^ T cells isolated from mouse splenocytes demonstrated an enhancement of CMI compared to that in mice immunized with PRRSV-KIV alone or PRRSV-KIV combined with IgM isotype control [27]. Therefore, these observations prompted us to investigate whether Mab-PR5nf1 could be used as a novel adjuvant to improve CMI in PRRSV-KIV immunization since previous reports suggested that normal PRRSV-KIV vaccination fails to elicit detectable induction of CMI in swine [21–23]. Consequently, a systematic comparison was conducted in this study to evaluate the protective efficacy of using PRRSV-specific IgM as an adjuvant for KIV against heterologous PRRSV. Moreover, inactivated classic PRRSV strain VR2332 and its attenuated live vaccine strain MLV were included for comparisons. The experimental protocol and immunization schedule are illustrated in Figure 1A, and the piglet groups are listed in Table 1. During vaccination, 100 μg of purified and inactivated PRRSV-VR2332 virion was used to immunize piglets, which were assigned to the normal KIV group. Additionally, inactivated PRRSV-VR2332 (100 μg) complexed with 1 mg of Mab-PR5nf1 was used to immunize piglets, which were assigned to the KIV + IgM group, whereas 1 mL of 1.0 × 10^6^ TCID_50_/mL MLV was administered to piglets that were assigned to the MLV control group. The first immunization for all groups started at −35 dpi. Serum samples from all immunized animals, including PBS-inoculated controls, were collected on a weekly basis before HP-PRRSV challenge and further subjected to ELISA screening for PRRSV-specific antibodies. A serum conversion result (a positive result indicating the presence of anti-PRRSV antibodies) was obtained for all vaccinated piglets, with no serum conversion observed for PBS controls (Fig. 1B). Taken together, these data indicated that all immunized piglets were successfully serum converted.

### IgM-adjuvanted inactivated PRRSV vaccine protected piglets against lethal challenge with HP-PRRSV

**Fig. 2:**
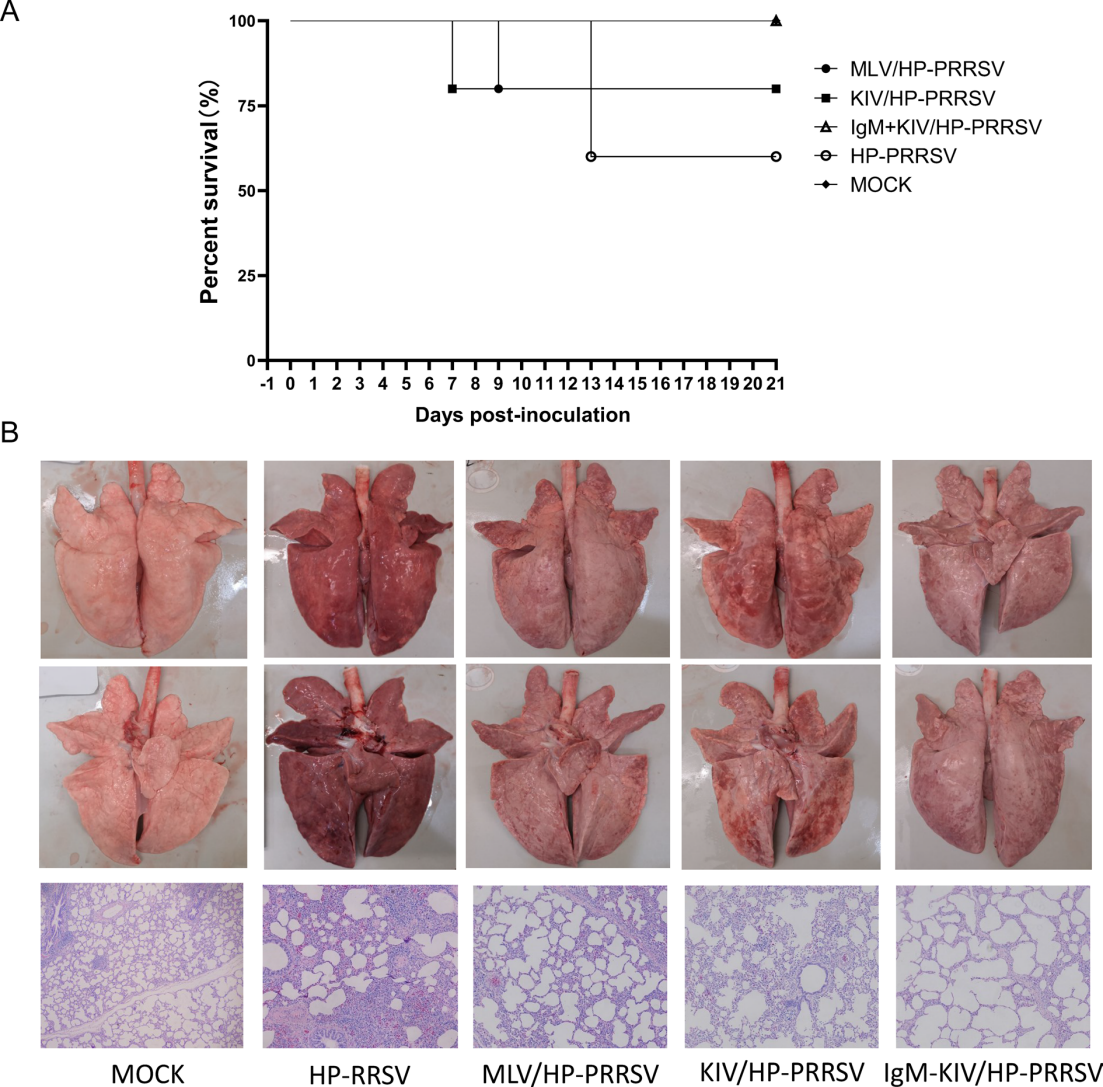
**IgM-adjuvanted inactivated PRRSV vaccine demonstrated improved protection of piglets against heterologous HP-PRRSV challenge**. **A.** All vaccinated animals or nonvaccinated controls were inoculated with 1.0 × 10^5^ TCID_50_ of HP-PRRSV-XJA1 via both intramuscular and intranasal routes. Clinical signs and survival rates were monitored daily for a total of 21 days. **B.** Representative ventral and dorsal lung images from surviving piglets of each group were captured immediately after piglets were necropsied at 21 dpi. Tissue samples from the lungs of all the animals necropsied at 21 dpi were fixed in 10% neutral buffered formalin, embedded in paraffin blocks and sectioned for histological analysis. The sections were stained with haematoxylin and eosin (H&E) to facilitate the observation of micropathological changes.

After immunization, all vaccinated piglets and a group of nonvaccinated control piglets were inoculated with HP-PRRSV strain XJA1 (HP-PRRSV) via both intramuscular and intranasal administration routes to ensure successful infection. Since traditional PRRSV-MLVs confer only partial or no protection against heterologous PRRSV isolates [12, 13], a heterologous HP-PRRSV was used for the challenge to observe whether using PRRSV-specific IgM as an adjuvant could improve vaccine efficiency. After HP-PRRSV challenge, mortality due to HP-PRRSV-XJA1 infection emerged in the KIV-vaccinated group at 7 dpi, with one piglet that died, but 4 piglets survived until the end of the experiment (Fig. 2A). Similar to the KIV group, one piglet died in the MLV group at 9 dpi, with 4 piglets surviving until the end of the experiment (Fig. 2A). However, two piglets died at 13 dpi in the nonvaccinated group, and the remaining animals survived (Fig. 2A). The overall survival rate of the KIV and MLV immunization groups (80%) was higher than that of the nonvaccinated group (60%), suggesting partial protection. However, it appears that KIV and MLV immunization may exacerbate PRRSV-induced symptoms and accelerate death in certain piglets during HP-PRRSV challenge. In contrast, the survival rate was 100% in the KIV + IgM group after challenge. Therefore, based on survival rates, IgM-adjuvanted PRRSV-KIV truly demonstrated improved protection efficiency against HP-PRRSV challenge and was even better than MLV. Afterwards, all surviving piglets were necropsied at 21 dpi to evaluate clinical signs. As shown in Fig. 2B, light-coloured, air-filled lungs were observed in the mock group. In contrast, lung lesions in unvaccinated piglets after challenge demonstrated extensive pneumonia, and microscopic examination revealed classic interstitial pneumonia with characteristic multifocal thickening in alveolar septa and spaces, as well as collapse of alveolar cells and serous exudation within bronchioles or alveolar spaces (Fig. 2B). However, lungs of pigs in the KIV groups demonstrated alleviation of pneumonia, with intermediate levels of pathological changes (Fig. 2B), whereas lungs of PRRSV-MLV-vaccinated piglets had only slight pathological changes. Similar to the MLV group, the IgM + KIV group demonstrated significant amelioration of pneumonia. Therefore, these data indicated that IgM-adjuvanted PRRSV-KIV immunization conferred better protection than either PRRSV-MLV or KIV immunization during heterologous HP-PRRSV challenge.

### IgM-adjuvanted inactivated PRRSV vaccine reduces viral shedding and results in improved viremia levels after HP-PRRSV challenge

**Fig. 3:**
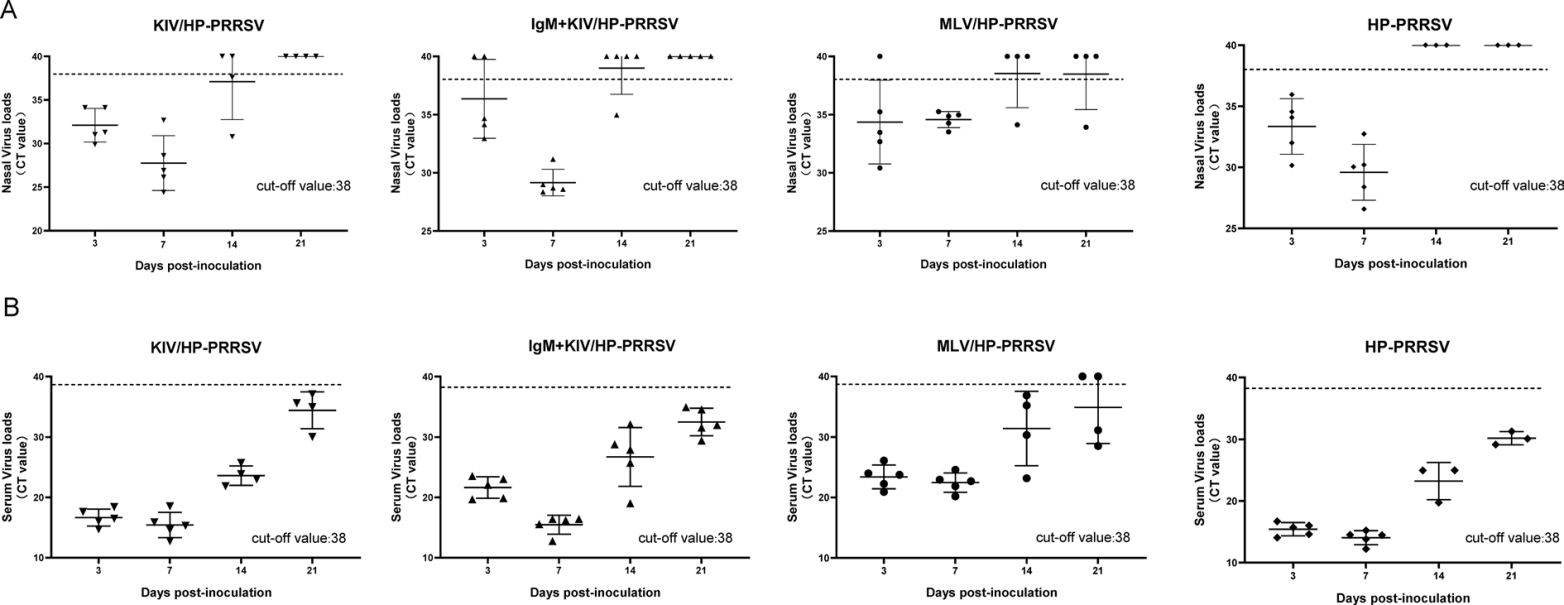
**Piglets immunized with IgM-adjuvanted inactivated PRRSV vaccine demonstrated improved viral shedding after heterologous HP-PRRSV challenge**. **A.** Nasal swab samples were collected at the indicated time points and harvested using TRIzol reagent for PRRSV RNA detection using IDEXX RealPCR PRRSV-2 RNA Mix. The Ct value of each sample is presented for comparison and was based on the manufacturer cut-off Ct value of 38. **B.** Serum samples were collected from each piglet at the indicated time points and processed using TRIzol reagent for PRRSV RNA detection.

To further investigate the factors involved in the improvement of protection efficiency in the IgM + KIV group, the viremia level and viral shedding level were examined using qPCR. As demonstrated in Fig. 3A, all piglets from the KIV-immunized group and nonvaccinated group demonstrated viral shedding at 3 and 7 dpi, as determined by the detection of viral RNA in nasal swabs. However, two piglets and one piglet from the KIV + IgM group and MLV group were negative for viral shedding at 3 dpi, respectively. Moreover, although the CT value of samples from the IgM + KIV group at 7 dpi was lower than that of samples from the MLV group (Fig. 3A), all piglets from the IgM + KIV group stopped viral shedding at 21 dpi, while one nasal swab sample from the MLV group remained positive for PRRSV RNA. In addition to viral shedding, the viremia level of piglets from different groups was examined. As demonstrated in Fig. 3B, at 3 dpi, the viremia level of the IgM + KIV and MLV groups was similar, but the CT value was higher than that of the KIV group, whereas the HP-PRRSV challenge group demonstrated the highest viremia (lowest CT value). However, at 7 dpi, the viremia levels of the KIV group and IgM + KIV group were similar and higher than those of the MLV group. At 14 dpi, although the IgM + KIV group demonstrated an improved trend of viremia compared with that of the KIV group, all piglets from the IgM + KIV group were still positive for PRRSV RNA at 21 dpi. In contrast, two surviving piglets from the MLV groups were negative for PRRSV viremia at 21 dpi (Fig. 3B).

**Fig. 4:**
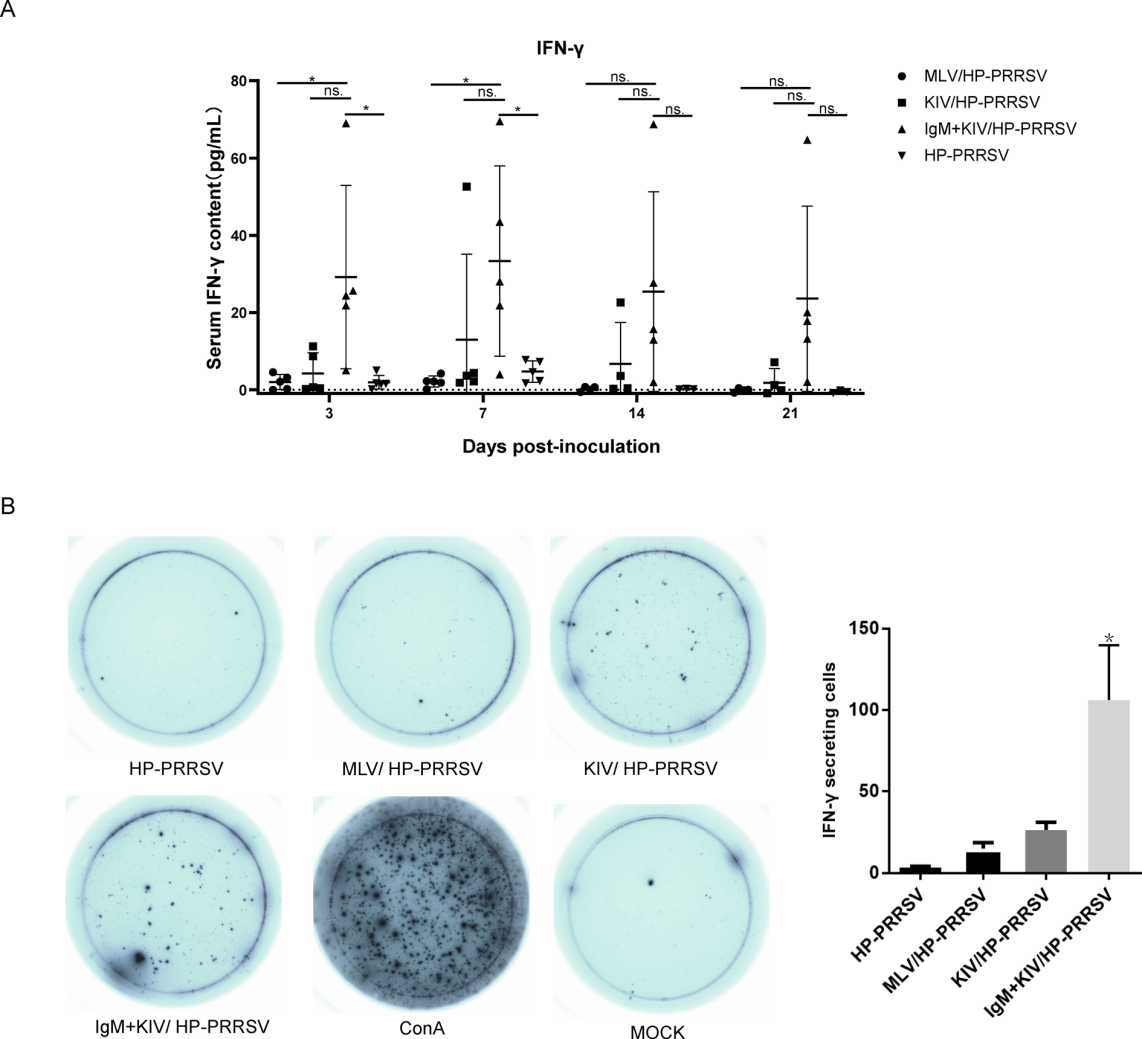
**IgM-adjuvanted inactivated PRRSV vaccine enhanced the secretion of IFN-γ after heterologous HP-PRRSV challenge**. **A.** After HP-PRRSV challenge, serum samples were collected at the indicated time points to examine serum IFN-γ levels using ELISA. All experiments were repeated at least three times for each serum sample. **B.** Peripheral blood mononuclear cells (PBMCs) were isolated from surviving piglets at the end of the experiment. Next, 1 × 10^4^ PBMCs were stimulated with 1 μg of recombinant PRRSV N proteins for 48 h. IFN-γ-producing cells were analysed by porcine IFN-γ ELISpot. PBMCs obtained from the mock piglets were treated with ConA or left untreated as positive and negative controls for the assay. The represented dot images were selected, and the numbers of dots for surviving piglets were recorded using an EliSPot reader. Data are expressed as the mean ± SD and were subjected to Student’s *t* test. Significant differences between the indicated groups are marked by “*” (*p* < 0.05), whereas “ns” means not significant.

It was confirmed very early that PRRSV infection inhibits IFN-γ production and MHC class I-mediated antigen presentation [33, 34], which contributes to the inhibited activation of cytotoxic T cells. Therefore, we further examined the serum IFN-γ level in serum samples. Our data suggested that serum IFN-γ levels in the HP-PRRSV-infected groups were extremely low except at 7 dpi after challenge. Similarly, a low level of IFN-γ was also observed in the MLV group, suggesting that PRRSV infection strongly inhibits the CTL response. However, it was notable that the KIV + IgM vaccination groups demonstrated the highest elevation of serum IFN-γ levels in piglets after HP-PRRSV challenge among all groups. This observation implied that the IgM-based immune complex (IgM-IC) may be preferentially taken up by dendritic cells (DCs) via FcμR for antigen presentation; therefore, PRRSV-KIV could effectively activate CD8^+^ T cells through the antigen cross-presentation capability of DCs. Conversely, we also noted that the serum IFN-γ level in MLV-immunized piglets after challenge was similar to that in HP-PRRSV-infected piglets (Fig. 4A). Although the survival rate in the IgM + KIV group was better than that in the MLV group, the viremia level in the MLV group was lower than that in the KIV + IgM group, which was inconsistent with the higher level of serum IFN-γ. Moreover, although IFN-γ levels in individual piglet of IgM + KIV group were highly variable and no significant reduction in viremia was observed in IgM + KIV group compared with that in MLV group (Fig. 4A), it was notable that the piglets of IgM + KIV group with the highest IFN levels demonstrated the lowest viremia, whereas piglet with the lowest IFN-γ levels demonstrated the highest viremia. These data suggested that the IgM-IC may contribute to the production of higher levels of IFN-γ and benefit viremia control. In addition to serum IFN-γ quantification, PBMCs isolated from surviving piglets were further subjected to a porcine IFN-γ ELISpots assay to evaluate the Th1-type response. During recombinant expression of PRRSV-all PRRSV structural proteins, only the PRRSV N protein was soluble in PBS and could be used for treating cells. Consequently, recombinant PRRSV N protein was used to treat PBMCs as previously described [33]. As demonstrated in Fig. 4B, after stimulation with recombinant PRRSV N protein, PBMCs from piglets of the IgM + KIV group demonstrated the highest numbers of IFN-γ-producing cells among all groups of animals, further supporting that the IgM-IC may contribute to the Th1 response during KIV immunization and lead to the highest level of IFN-γ production after HP-PRRSV challenge.

**Fig. 5:**
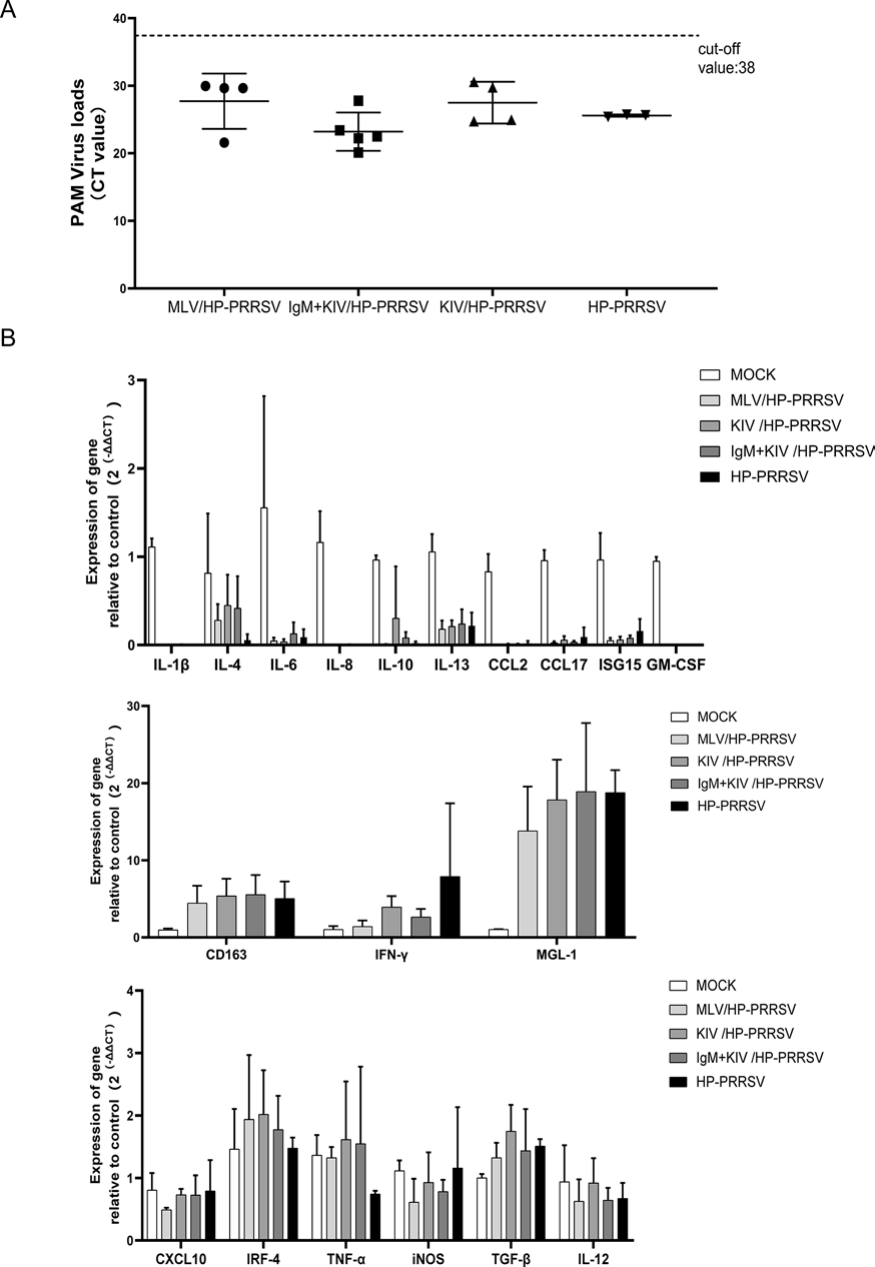
**Evaluation of PRRSV RNA and gene expression alterations in alveolar macrophages from surviving piglets in different groups**. **A.** RNA from PAMs collected from necropsied piglets at 21 dpi was harvested using TRIzol reagent and further subjected to PRRSV RNA detection using IDEXX RealPCR PRRSV-2 RNA Mix. The Ct value of each sample is presented for comparison and was based on the manufacturer cut-off Ct value of 38. **B.** RNA from PAMs was harvested using TRIzol reagent for RNA extraction and reverse transcription. The expression of macrophage activation-related genes (M1 or M2) was examined by qPCR for the indicated genes. All experiments were repeated at least three times. Data are expressed as the mean ± SD.

In addition to evaluation of viral shedding and viremia, PAMs were also collected from all surviving piglets at the end of the experiment and subjected to qPCR analysis of PRRSV RNA. Notably, all PAMs were positive for PRRSV RNA (Fig. 5A). This result suggested that a low level of PRRSV replication was still ongoing, as some piglets from the MLV group stopped viral shedding and showed a clearance of viremia (Figs. 3A and B, Fig. 5A). Afterwards, to further understand the functionality of PAMs in the different groups, genes representing macrophage function were investigated, such as M1-like cytokines (TNF-α, IL-1β, IL-6, IL-12, IFN-γ, iNOS, TGF-β, IRF-4, and CCL17) and M2-like cytokines (IL-4, IL-10, CD163, IL-13, and MGL-1) [35, 36], as well as the chemokines CCL2 and CXCL10 and antiviral genes such as ISG15 representing the type I IFN pathway. Based on our results, except for CD163, IFN-γ and MGL-1, the expression of most genes (IL-1β, IL-4, IL-6, IL-8, IL-10, IL-13, CCL-2, CCL-17, ISG15 and GM-CSF), regardless of M1 or M2 category, was significantly downregulated after HP-PRRSV challenge, and no significant difference in expression levels was observed for these genes among the different groups except the mock group (Fig. 5B). However, it is worth noting that MGL-1 expression was significantly increased in all surviving piglets after HP-PRRSV challenge at 21 dpi (Fig. 5B), implying a potential role of MGL-1 during PRRSV infection, but this hypothesis requires further investigation. Conversely, no significant difference in gene expression levels was observed between the mock group and the other groups for M1-related genes (IRF4, TNF-α, iNOS, TGF-β, and IL-12) and CXCL10 (Fig. 4B). These data suggested that PRRSV still replicated inside PAMs of surviving piglets and might regulate host gene expression in PAMs at 21 dpi. Taken together, the above data suggested that IgM + KIV immunization in piglets could reduce viral shedding and improve viremia, as well as enhance the host IFN-γ response after HP-PRRSV challenge.

### Inactivated PRRSV and MLV immunization induced different antibodies profiles

**Fig. 6:**
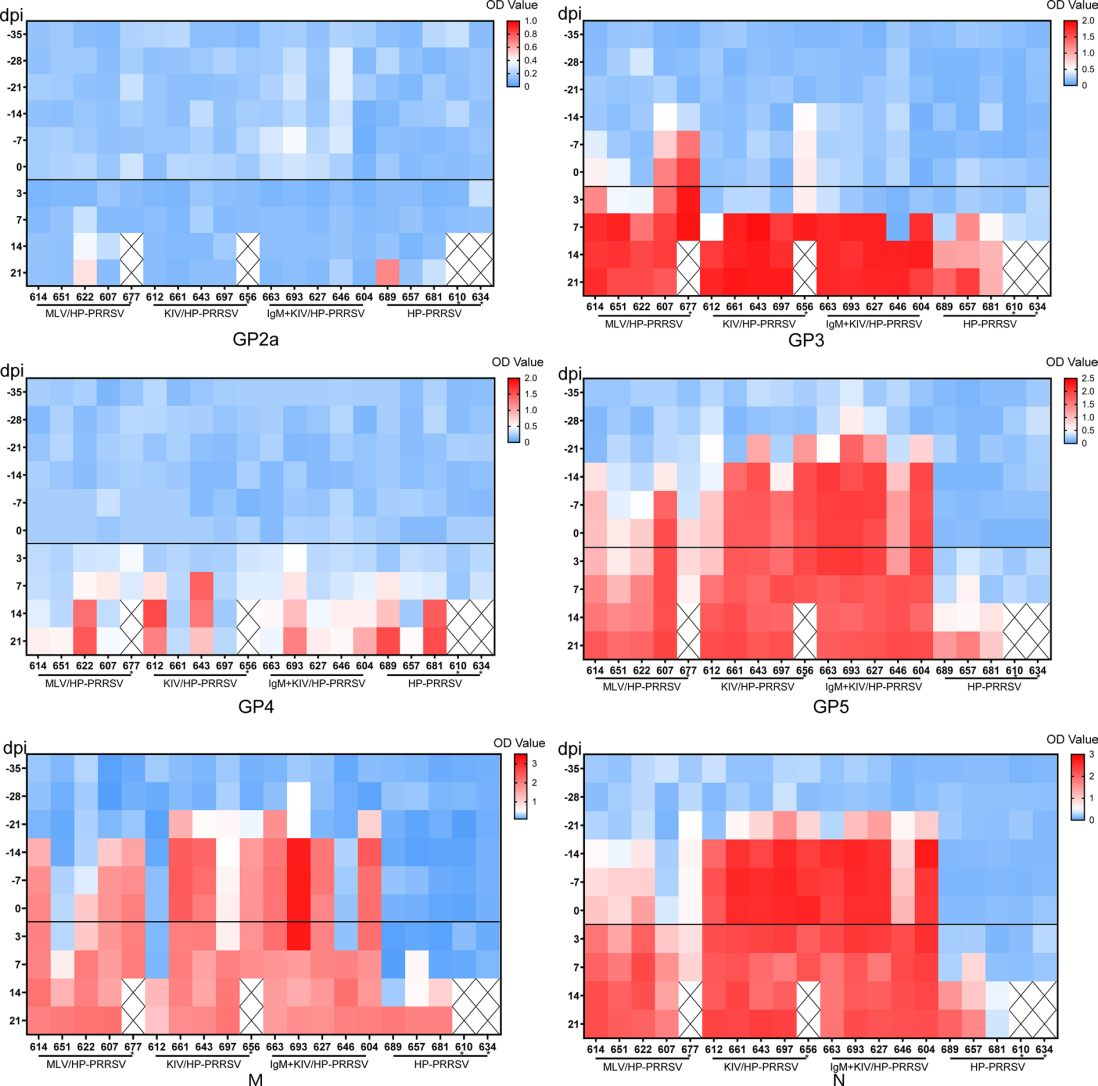
**Antibody profiling of piglet serum against different PRRSV structural proteins.** All serum samples of different groups of piglets collected from the indicated time points were subjected to indirect ELISA to measure antibody levels against all PRRSV structural proteins, including the GP2a, GP3, GP4, GP5 M and N proteins. The absolute OD 450 value was used to generate a heatmap for visualization of antibody profiles against PRRSV structural proteins. Deceased piglets are marked by “*”.

In addition to IFN-γ quantification, to further understand the factors involved in the improvement of PRRSV-KIV immunization efficiency by IgM, all PRRSV structural proteins (SPs) were expressed in *E. coli* and purified for ELISA analysis. Serum samples of different groups throughout the entire experiment were investigated to understand the dynamics of PRRSV-specific antibodies against different SPs. The expressed and purified PRRSV SPs (Additional file 1), including GP2a, GP3, GP4, GP5, M and N, were subjected to SDS‒PAGE-based analysis of purity (Additional file 1). Next, all these SPs were used to coat ELISA plates. Based on the results, it appeared that most antibodies whose production was evoked by KIV and KIV + IgM immunization before HP-PRRSV challenge were mainly restricted to recognition of the GP5, N and M proteins (Fig. 6). Similarly, an early onset of the production of GP5-, M- and N-specific antibodies was observed in both the KIV and KIV + IgM immunization groups. In contrast, antibodies against GP3 could be detected in one piglet 3 weeks (−14 dpi) after MLV immunization, with four piglets positive for anti-GP3 antibodies before challenge, whereas only one piglet from the KIV-immunized group appeared to be positive for GP3-specific antibodies. Moreover, GP2a- and GP4-specific antibodies were not detectable in all animals before challenge regardless of the vaccine types used for immunization. Afterwards, production of GP4-specific antibodies was found to be evoked by HP-PRRSV challenge in the MLV and KIV + IgM groups (Fig. 6), while two surviving piglets from the KIV-immunized groups remained negative for anti-GP4 antibodies throughout the study. Additionally, one piglet from the MLV group and another one from the HP-PRRSV-challenged group were positive for anti-GP2a antibodies. Combined with the viremia results, it appears that higher levels of GP5- and M-specific antibodies in the KIV and KIV + IgM groups did not reduce viremia, suggesting that GP5 and M may not be the only determinants for PRRSV virion neutralization. In contrast, the onset of the production of GP3- and GP4-specific antibodies at 7 dpi appeared to correlate with a reduction in viremia in the KIV and KIV + IgM groups, whereas a low level of viremia at 3 dpi was observed in the MLV group with the presence of anti-GP3 antibodies. Moreover, viremia in piglets of the MLV group continued to decline at 7 dpi, and GP4-specific antibodies simultaneously appeared. These data suggested that GP3- and GP4-specific antibodies might contribute to the neutralization and clearance of PRRSV from the circulation. Furthermore, our data also suggested that KIV + IgM immunization accelerated the development of GP4-specific antibodies in piglets after HP-PRRSV challenge, whereas two piglets in the KIV-immunized group remained negative for GP4-specific antibodies at 21 dpi. Taken together, these data suggested that PRRSV-KIV and MLV immunization induced different antibody profiles against various PRRSV structural proteins. Application of PRRSV-specific IgM as a KIV adjuvant does not cause significant alteration of host antibody profiles during immunization but improves the antibody response to GP4 after HP-PRRSV challenge.

### The IgM-adjuvanted inactivated PRRSV vaccine induced the production of higher levels of neutralizing antibodies than the conventional inactivated PRRSV vaccine after challenge

**Fig. 7:**
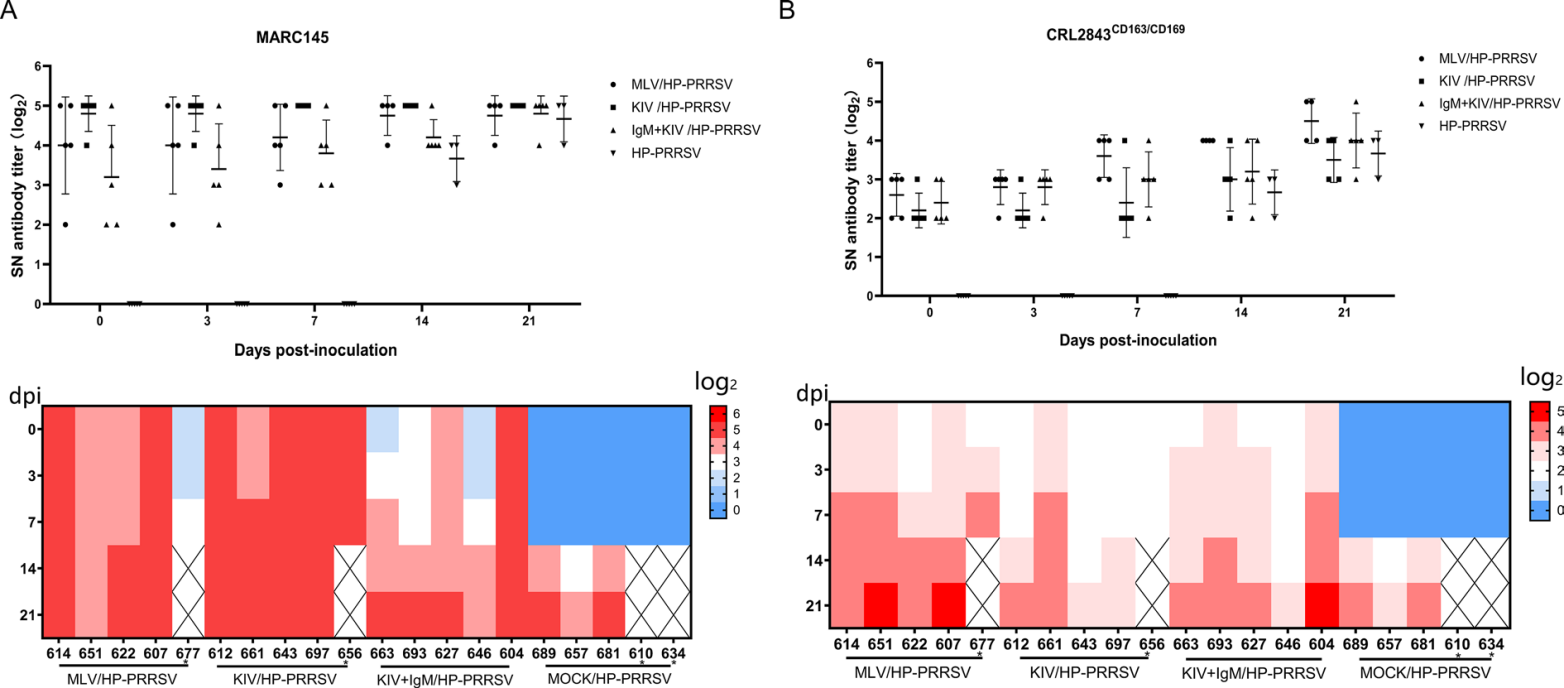
**Evaluation of PRRSV-neutralizing antibodies against the HP-PRRSV-JXA1 strain. A.** Serum samples from piglets of different groups were collected at the indicated dpi after HP-PRRSV challenge. Sera were further tested by neutralization assays using twofold serial dilutions to evaluate virus-neutralizing activity against HP-PRRSV infection in MARC-145 cells. Data are expressed as the mean ± SD. The maximum fold dilution of the serum neutralization titre from individual piglets determined in MARC-145 cells was used to generate a heatmap for visualization of neutralization titre kinetics for each piglet throughout the whole experiment. **B**. Serum samples from piglets of different groups were collected at the indicated dpi after HP-PRRSV challenge. Sera were further tested by neutralization assays using twofold serial dilutions to evaluate virus-neutralizing activity against HP-PRRSV infection in CRL-2843^CD163/CD169^ cells. Data are expressed as the mean ± SD. The maximum fold dilution of the serum neutralization titre from individual piglets determined in CRL-2843^CD163/CD169^ cells was used to generate a heatmap for visualization of neutralization titre kinetics for each piglet. Deceased piglets are marked by “*”.

The above data suggested that PRRSV-KIV and MLV immunization induced a different antibody profile against PRRSV structural proteins, whereas application of IgM as a KIV adjuvant did not change antibody profiles for PRRSV SPs during immunization but caused a certain difference after HP-PRRSV challenge. Consequently, we further evaluated serum virus-neutralizing (VN) antibody levels against HP-PRRSV from different groups in MARC-145 cells, so the potential roles of VN antibodies during KIV- and MLV-induced protection against HP-PRRSV challenge could be revealed. However, unexpectedly, the VN titre of 4 piglets in the KIV group reached 32-fold before HP-PRRSV challenge, whereas the VN titres of all five piglets in the KIV group reached 32-fold at 7 dpi (Fig. 7A). In contrast, only 2 and 1 piglets from the MLV and IgM + KIV groups, respectively, demonstrated VN titres of 32. At the later time point (21 dpi), an increase in VN titres was observed in these two groups (Fig. 7A). Nevertheless, there was still one surviving piglet in the MLV group and one piglet in the IgM + KIV group that demonstrated VN titres below 32 (Fig. 7A).

In our previous study investigating the inhibition of PRRSV virion attachment using polyethyleneimine (PEI) [37], we noticed that PAMs were highly susceptible to PRRSV virion binding and that the binding capacity of PRRSV virions to PAMs was nearly 100-fold higher than that of PAMs to MARC-145 cells [37]. Therefore, inconsistencies in PRRSV VN titres and vaccine protection efficiency in different groups implied that serum VN titres evaluated in MARC-145 cells in vitro may not reflect the authentic VN titre of serum in vivo. However, as primary PAMs are suspended cells and not suitable for the VN assay, we created a novel PRRSV permissive cell line based on immortalized PAMs, CRL-2843 bearing porcine CD163, by further introducing CD169 to increase PRRSV susceptibility (Additional file 2). Based on our data, although original CRL-2843 cells were nonpermissive for PRRSV, introduction of CD163 conferred susceptibility to PRRSV in CRL-2843 cells (Additional file 2C). Co-expression of CD163 and CD169 further enhanced PRRSV susceptibility in CRL-2843^CD163/CD169^ stable cells (Additional file 2C). Consequently, the VN titres of piglet serum from all groups were re-evaluated in CRL-2843^CD163/CD169^ cells. As demonstrated in Fig. 7B, before HP-PRRSV challenge, only one serum sample from the KIV group showed a VN titre of 8 in CRL-2843^CD163/CD169^ cells, whereas the VN titres of the other 4 piglets in the KIV group were 4. In contrast, 3 samples from the MLV group and 2 samples from the IgM-KIV group demonstrated a VN titre of 8 in CRL-2843^CD163/CD169^ cells. After HP-PRRSV challenge, a rapid increase in serum VN titres was observed in both the MLV and IgM-KIV groups, as determined in CRL-2843^CD163/CD169^ cells (Fig. 7B). At 14 dpi, all 4 surviving piglets in the MLV group and 2 piglets from the IgM-KIV group demonstrated VN titres of 16, whereas only one piglet from the KIV group demonstrated a VN titre of 16 (Fig. 7B). At 21 dpi, on the one hand, all 4 surviving piglets in the MLV group and 4 piglets from the IgM-KIV group demonstrated VN titres higher than 16, with 2 surviving piglets in the MLV group and 1 piglet from the IgM-KIV group demonstrating VN titres of 32. On the other hand, only 2 surviving piglets from the KIV group demonstrated a VN titre of 16 in CRL-2843^CD163/CD169^ cells (Fig. 7B). Overall, compared with VN titres determined in MARC-145 cells (Fig. 7A), the serum VN titres determined in CRL-2843^CD163/CD169^ appeared to be more consistent with viremia data since 4 surviving piglets from the MLV group demonstrated lower viremia levels and had higher VN titres (determined in CRL-2843^CD163/CD169^). Moreover, although no significant difference could be observed for antibody levels against different PRRSV SPs between the KIV and IgM + KIV groups before HP-PRRSV challenge, VN titres in the IgM + KIV group appeared to be higher than those in the KIV group after HP-PRRSV challenge, as determined in CRL-2843^CD163/CD169^ cells. These data suggested that the application of IgM as an adjuvant to form immune complexes might improve antigen uptake by antigen-presenting cells and enhance the host adaptive antibody response against PRRSV. In conclusion, our data suggested that PRRSV-specific IgM could be applied as a novel adjuvant to improve the protection efficiency of inactivated PRRSV vaccines against HP-PRRSV challenge.

## Discussion

Since the discovery of PRRSV, several attenuated vaccines have been developed against both PRRSV genotypes and licenced in different countries based on the particular circulating genotypes in each country. However, safety has been a major concern for PRRSV-MLVs for a long time [12, 16]. In contrast, due to poor efficacy, inactivated PRRSV vaccines (KIV) cannot meet practical needs unless significant improvements can be made [12]. In the present study, our results demonstrated that mouse-derived Mab-PR5nf1 maintains its biological activity in swine and that Mab-PR5nf1-adjuvanted PRRSV-KIV yielded improved protective efficacy (100% survival rate after HP-PRRSV challenge) when compared with normal PRRSV-KIV- and MLV (80% survival rate after HP-PRRSV challenge). Although antigen-specific IgM was shown to enhance vaccine-induced immunity during murine malaria vaccination as early as the 1980s [38], the application of IgM as a novel vaccine adjuvant has never been tested in virus vaccination. In this study, we conducted a systematic investigation to utilize PRRSV-specific IgM as a novel adjuvant for PRRSV-KIV to boost host immunity against heterologous HP-PRRSV challenge.

Theoretically, IgM as a vaccine adjuvant confers several advantages due to unique features of IgM during immunization [39], such as enhanced B-cell receptor signalling or enhanced antibody responses via a process known as antibody-mediated feedback regulation [40, 41]. Moreover, a recent report demonstrated that Fc receptor for IgM (FcμR) serves as a costimulatory molecule for T-cell activation [42], which implies that the application of antigen-specific IgM as a vaccine adjuvant may enhance the activation of T cells [42]. Consistent with these observations, our preliminary report demonstrated that immunization of mice with a cocktail consisting of Mab-PR5nf1, inactivated HP-PRRSV-SD16 virus and normal vaccine adjuvant significantly enhanced cell-mediated immunity (CMI), as evidenced by PRRSV-specific IFN-γ ELIspot for mouse CD8^+^ T cells [27], whereas an IgM isotype control antibody did not confer enhanced CMI [27]. These data suggest that IgM-mediated enhancement of IFN-γ-secreting CD8^+^ T cell levels during vaccination was antigen specific, which might require direct binding between IgM and PRRSV virions to form the IgM immune complex (IgM-IC). Therefore, IgM Mab-PR5nf1 might serve as a novel adjuvant for any PRRSV strain due to its broad reactivity with heterologous PRRSV strains.

It was observed very early that PRRSV-specific T cells enumerated by IFN-γ ELISpot did not appear until 2 weeks after PRRSV inoculation, and their abundance exhibited substantial variation over time and among animals [33]. This observation was consistent with a later report demonstrating that PRRSV NSP1α targets Swine Leukocyte Antigen Class I (SLA-I) molecules for proteasomal degradation to inhibit the activation of cytotoxic T cells [34]. Similarly, the level of serum IFN-γ in the HP-PRRSV-infected groups was extremely low except at 7 dpi, even in MLV-immunized piglets, indicating that PRRSV infection strongly inhibited the CTL response. However, it was notable that the KIV + IgM group demonstrated the highest serum IFN-γ levels in piglets after HP-PRRSV challenge among all vaccinated groups. These data implied that the IgM-based immune complex (IgM-IC) may be preferentially taken up by dendritic cells (DCs) via FcμR for antigen presentation; therefore, PRRSV-KIV could potentially activate CD8^+^ T cells through the antigen cross-presentation capability of DCs. Moreover, a recent report suggested that FcμR serves as a costimulatory molecule for T-cell activation [42], implying that the IgM-IC may enhance the activation of T-cell activation during PRRSV-KIV immunization [42]. The Fc receptor for IgM (FcμR) is a transmembrane protein initially referred to as “Fas apoptosis inhibitory molecule 3” (FAIM3) or TOSO [43], which was proposed to inhibit Fas/CD95-induced apoptosis in T cells [43, 44]. However, FcμR-deficient mice display dysregulated functions of neutrophils, dendritic cells, and B cells [45], as well as defects in the maturation and differentiation of dendritic cells [46]. Therefore, FcμR may be crucial for the effective activation of the immune system and the development of an adaptive immune response. Although swine FcμR has not been cloned and sequenced, a predicted sequence was deposited in GenBank, and we are currently working on the cloning of swine FcμR to further investigate its role during the development of antigen-specific CTLs.

In addition to the enhanced IFN-γ secretion of PRRSV-KIV + IgM-immunized piglets after challenge, we also noted that the serum IFN-γ level in MLV-immunized piglets after challenge was similar to that of HP-PRRSV-infected piglets. Although the survival rate in the KIV + IgM-immunized groups was better than that in the MLV-immunized groups, the viremia level in the MLV-immunized group was lower than that in the KIV + IgM group, which was not consistent with the higher serum IFN-γ level, as enhanced CTL responses should reduce viremia levels. A possible explanation is the difference in antigens between PRRSV-KIV and MLV during vaccination for stimulating an adaptive immune response. During KIV immunization, only PRRSV structural proteins (SPs) are presented by antigen-presenting cells (APCs) to stimulate an adaptive immune response for immune memory. However, during the MLV immunization and HP-PRRSV challenge phase, the majority of newly synthesized viral proteins in PRRSV-replicating cells should be PRRSV NSPs, which account for three-fourths of the protein-coding sequence across the PRRSV genome, and both neonatal PRRSV SPs and NSPs could be processed by endogenous antigen presentation pathways and finally presented by SLA-I molecules to activate corresponding CD8^+^ T cells or be recognized by activated PRRSV-specific CTLs. It is possible that the source of IFN-γ in the IgM-KIV group was effector T cells recognizing PRRSV SPs; however, the lack of CTLs recognizing PRRSV NSPs in the KIV or IgM + KIV groups might not confer full protection to effectively control viremia due to the narrower antigen range (restricted to PRRSV SPs) recognized by CTLs, although the IFN-γ level was higher in IgM + KIV-immunized piglets.

Neutralizing antibodies (NAs) against PRRSV have recently been considered to be an effective component of adaptive immunity against PRRSV [12, 47], since PRRSV-specific antibody kinetics suggest that the onset of the production of NAs after experimental infection in piglets correlates with virus clearance from the circulation and tissues [48, 49]. In our previous research evaluating a novel attenuated PRRSV vaccine strain, A2MC2-P90, piglets immunized with A2MC2-P90 rapidly developed a stronger protective humoral immune response, as evidenced by higher titres of neutralizing antibodies, more rapid clearance of viremia and less nasal virus shedding [50]. Moreover, administration of a PRRSV-specific neutralizing monoclonal antibody (IgG) resulted in a significant reduction in PRRSV-induced pulmonary pathological changes and viral loads in PAMs after PRRSV challenge [51], further supporting a protective role of NAs in PRRSV infection. As previous reports suggested that immunization of PRRSV-KIV cannot result in a detectable production of PRRSV-specific antibodies (neither non-NA nor NA) [23], it was surprising that KIV immunization alone in this study was capable of inducing the production of high-titre NAs (approximately 1:32), as determined in vitro in MARC-145 cells. Additionally, although one piglet from the KIV-immunized group died after challenge (80% survival rate), this result was better than that for the HP-PRRSV challenge group (60% survival), suggesting that the administration of KIV conferred a certain benefit after immunization. However, it was notable that 100 µg of highly purified PRRSV virions was used for immunization in this study; therefore, it was possible that the high dose and purification procedure significantly improved the antigenicity of PRRSV-KIV, thus leading to the induction of PRRSV-specific neutralizing antibodies. Conversely, our data also suggested that VN titres determined in MARC-145 cells (relatively higher for the KIV group but lower for the MLV group) were not consistent with the VN titres determined using the PAM-derived cell line CRL-2843^CD163/CD169^ (relatively lower for KIV but higher for MLV), whereas VN titres determined using the PAM-derived cell line CRL-2843^CD163/CD169^ appeared to be more correlated with viremia levels after HP-PRRSV challenge, especially for surviving piglets in the MLV group.

The mechanism of antibody-mediated PRRSV neutralization remains controversial, whereas conflicting data have been obtained from various studies [10, 52, 53]. Initially, GP5, the major glycosylated envelope protein encoded by PRRSV-ORF5, was considered to be a major target for PRRSV NAs based on observations of related viruses such as lactate dehydrogenase-elevating virus (LDV) and equine arteritis virus (EAV) [54]. However, this view has been challenged in recent years [55], and one report demonstrated that anti-PRRSV M-GP5 ectodomain-specific antibodies from PRRSV-neutralizing serum bound to the virus but did not neutralize it [56]. In contrast, it was demonstrated that other structural proteins, such as M protein (encoded by ORF6, an unglycosylated matrix protein) and minor glycoproteins (GP2, GP3, and GP4), possess neutralizing epitopes as well [54, 57–59]. Our study demonstrated that PRRSV-KIV and MLV vaccination evoked different antibody profiles against PRRSV SPs. KIV immunization mainly evoked a rapid production and higher level of antibodies against PRRSV N protein and GP5 protein, whereas MLV immunization mainly evoked antibodies against GP3 proteins. Moreover, levels of antibodies against GP2a and GP4 appeared to be minimal before HP-PRRSV challenge regardless of KIV or MLV immunization, but a slight evaluation in GP4-specific antibody levels was observed after HP-PRRSV challenge in the MLV group and IgM + KIV group. Therefore, the antibody response required for full protection against PRRSV challenge might require further analysis. Nevertheless, our data implied that antibody-mediated neutralization of PRRSV may not be determined by a single SP but rather involves multiple PRRSV SPs, such as GP3 and GP4.

In conclusion, in this research, we applied a novel PRRSV-specific IgM monoclonal antibody (Mab)-PR5nf1 as a novel adjuvant for the formulation of a cocktail composed of inactivated PRRSV and Mab-PR5nf1 along with a normal adjuvant to enhance the PRRSV-KIV vaccine. Our results suggested that the overall survival rate and cell-mediated immunity were significantly improved by adding PRRSV-specific IgM to the PRRSV-KIV vaccine and that the overall survival rate in the IgM + KIV group was even higher than that in the MLV group. Our data provide not only a new formula for the development of an effective PRRSV-KIV vaccine for practical use but also a novel method for improving antigen-specific CMI induction by inactivate vaccines and subunit vaccines.

## Supplementary Information

**Additional file 1: SDS‒PAGE analysis of recombinant PRRSV structural proteins.**

**Additional file 2:** **CRL-2843CD163/CD169cells were highly susceptible to PRRSV infection. A**. SDS‒PAGE analysis of recombinant CD163(CD163 N truncation, containing the first 5 SRCR domains) and CD169(CD169 N truncation, first 540 aa) proteins; **B**. Normal CRL-2843, CRL-2843CD163, and CRL-2843CD163/CD169cells and PAMs were harvested for SDS‒PAGE and Western blot analysis using rabbit anti-CD163 and anti-CD169 polyclonal antibodies. Tubulin was probed on the same membrane as the protein loading control. **C**. Normal CRL-2843, CRL-2843CD163, and CRL-2843CD163/CD169cells were infected with PRRSV(0.1 MOI) for 24 hand then harvested for Western blot analysis of the PRRSV N protein level. Tubulin was probed on the same sample as the protein loading control.

## Data Availability

The authors confirm that the data supporting the findings of this study are available within the article and its supplementary materials.

## Abbreviations

PRRSV: porcine reproductive and respiratory syndrome virus
ORFs: open reading frames
MLV: modified live virus
KIV: inactivated PRRSV vaccine
CMI: cell-mediated immunity
HP-PRRSV: highly pathogenic PRRSV
NAbs: neutralizing antibodies
mAbs: monoclonal antibodies
PAMs: porcine alveolar macrophages
SDS‒PAGE: sodium dodecyl sulfate‒polyacrylamide gel electrophoresis
qPCR: quantitative real-time PCR
BALF: bronchoalveolar lavage fluid
