# Supplementary figures and images for: A porcine reproductive and respiratory syndrome virus (PRRSV)-specific IgM as a novel adjuvant for an inactivated PRRSV vaccine improves protection efficiency and enhances cell-mediated immunity against heterologous PRRSV challenge
Source: Vet Res. 2022 Aug 19;53:65. doi: 10.1186/s13567-022-01082-5 (PMC9389807; doi:10.1186/s13567-022-01082-5)

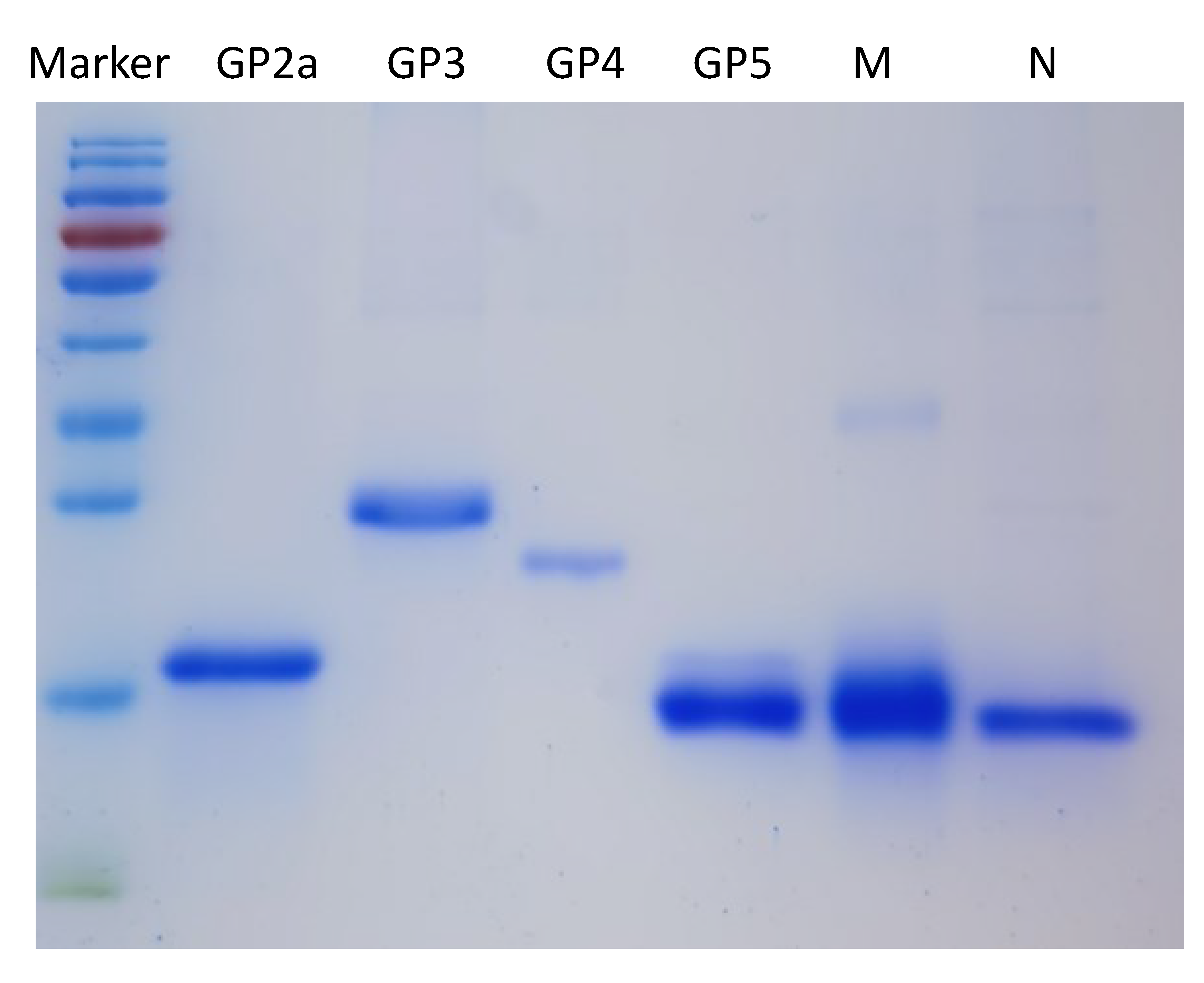

Supplement: Supplementary file 1 — Additional file 1: SDS‒PAGE analysis of recombinant PRRSV structural proteins. [file 13567_2022_1082_MOESM1_ESM.tiff]

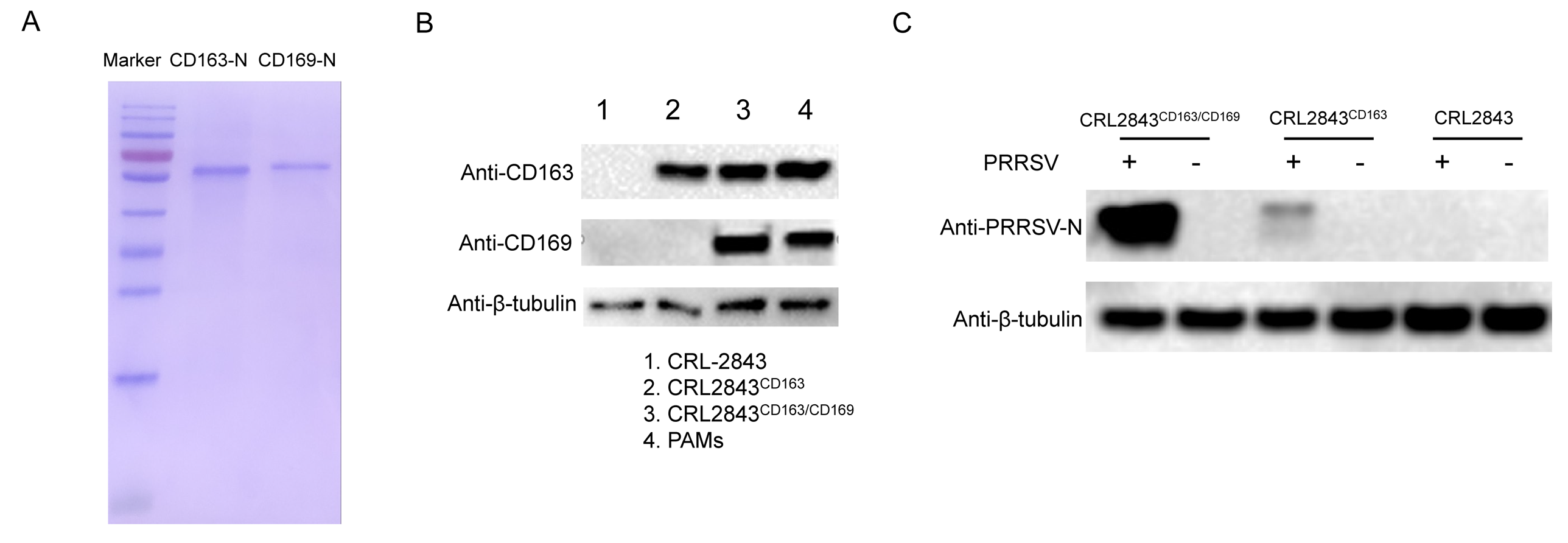

Supplement: Supplementary file 2 — Additional file 2: CRL-2843CD163/CD169cells were highly susceptible to PRRSV infection. A. SDS‒PAGE analysis of recombinant CD163(CD163 N truncation, containing the first 5 SRCR domains) and CD169(CD169 N truncation, first 540 aa) proteins; B. Normal CRL-2843, CRL-2843CD163, and CRL-2843CD163/CD169cells and PAMs were harvested for SDS‒PAGE and Western blot analysis using rabbit anti-CD163 and anti-CD169 polyclonal antibodies. Tubulin was probed on the same membrane as the protein loading control. C. Normal CRL-2843, CRL-2843CD163, and CRL-2843CD163/CD169cells were infected with PRRSV(0.1 MOI) for 24 hand then harvested for Western blot analysis of the PRRSV N protein level. Tubulin was probed on the same sample as the protein loading control. [file 13567_2022_1082_MOESM2_ESM.tiff]
